# Characterization of a *Lactiplantibacillus plantarum* R23 Isolated from Arugula by Whole-Genome Sequencing and Its Bacteriocin Production Ability

**DOI:** 10.3390/ijerph18115515

**Published:** 2021-05-21

**Authors:** Joana Barbosa, Helena Albano, Beatriz Silva, Maria Helena Almeida, Teresa Nogueira, Paula Teixeira

**Affiliations:** 1 CBQF-Centro de Biotecnologia e Química Fina–Laboratório Associado, Escola Superior de Biotecnologia, Universidade Católica Portuguesa, 4169-005 Porto, Portugal; microhel@gmail.com; 2 Colégio de São Gonçalo, 4600-014 Amarante, Portugal; beatriz_silva_22@hotmail.com; 3 Colégio Internato dos Carvalhos, 4415-133 Pedroso, Portugal; mhelenocas.almeida@gmail.com; 4 Instituto Nacional de Investigação Agrária e Veterinária, I.P., 2780-157 Oeiras and 4485-655 Vairão, Portugal; teresa.nogueira@iniav.pt; 5 cE3c-Centre for Ecology, Evolution and Environmental Changes, Faculdade de Ciências da Universidade de Lisboa, 1749-016 Lisboa, Portugal

**Keywords:** bio-preservation, Coagulin A, food safety, pediocin, plantaricin, starter culture

## Abstract

*Lactiplantibacillus plantarum* is one of the lactic acid bacteria species most used as probiotics and starter cultures in food production. Bacteriocin-producers *Lpb. plantarum* are also promising natural food preservatives. This study aimed to characterize *Lpb. plantarum* R23 and its bacteriocins (R23 bacteriocins). The genome sequence of *Lpb. plantarum* R23 was obtained by whole-genome sequencing (WGS) in an Illumina NovaSeq platform. The activity of *Lpb. plantarum* R23-produced bacteriocin against two *Listeria monocytogenes* strains (L7946 and L7947) was evaluated, and its molecular size was determined by tricine-SDS-PAGE. No virulence or antibiotic resistance genes were detected. Four 100% identical proteins to the class II bacteriocins (Plantaricin E, Plantaricin F, Pediocin PA-1 (Pediocin AcH), and Coagulin A) were found by WGS analysis. The small (<6.5 kDa) R23 bacteriocins were stable at different pH values (ranging from 2 to 8), temperatures (between 4 and 100 °C), detergents (all, except Triton X-100 and Triton X-114 at 0.01 g/mL), and enzymes (catalase and α-amylase), did not adsorb to the producer cells, had a bacteriostatic mode of action and their maximum activity (AU/mL = 12,800) against two *L. monocytogenes* strains occurred between 15 and 21 h of *Lpb. plantarum* R23 growth. *Lactiplantibacillus plantarum* R23 showed to be a promising bio-preservative culture because, besides being safe, it produces a stable bacteriocin or bacteriocins (harbors genes encoding for the production of four) inhibiting pathogens as *L. monocytogenes*. Further studies in different food matrices are required to confirm this hypothesis and its suitability as a future starter culture.

## 1. Introduction

Adequate amounts of ready-to-eat vegetables should be consumed daily as part of a diversified and healthy diet because they are an essential source of vitamins, minerals, dietary fiber, and phytonutrients [1]. Additionally, fresh vegetable consumption also improves gut bacteria diversity, contributing to improved metabolisms [1].

Despite the constant considerable contamination of ready-to-eat vegetables with important foodborne pathogens [2,3,4], bacteria with benefits for the food industry, such as lactic acid bacteria (LAB), have also been found in these products [5,6]. *Lactiplantibacillus plantarum* (previously *Lactobacillus plantarum*) [7] is one of the main promising species of LAB widely used in food production as a probiotic and starter culture [8,9]. Due to their long history of safe human use, most of the LAB genera, particularly the *Lactiplantibacillus* genus, are included in the qualified presumption of safety (QPS) recommendation of the European Food Safety Authority [10]. Their ingestion has been suggested to confer various health benefits, including modulating the immune system, and increasing and improving resistance to malignant and infectious diseases [11,12].

Several LAB were reported to produce a wide range of antimicrobial substances that comprise organic acids, diacetyl, hydrogen peroxide, and bacteriocins [13,14,15]. The most studied bacteriocins are those produced by LAB, and many of these have shown considerable potential as food preservatives [16] or as therapeutic or bio-controlling agents [17,18,19,20]. The added value of their use is due to their non-toxic nature, specific action, physical stability, and high antimicrobial activity [21,22,23,24].

In recent years, many bacteriocins have been characterized, but nisin (NisaplinTM) is the only commercially available bacteriocin approved by the Food and Drug Administration [25]. This can be circumvented, because to exert their bioprotective role, bacteriocins can be incorporated into food by using bacteriocin-producing LAB in situ rather than as an ingredient [26].

This study aimed to complement the characterization of a previously isolated bacteriocin-producing *Lactiplantibacillus plantarum* R23 [15] and its bacteriocins by whole-genome sequencing analysis. This is a useful process to predict the virulent and resistant phenotypes of bacterial strains. Besides its safety and bacteriocin gene identification, the in vitro characterization, mode of action, and stability in different environments of its produced bacteriocin(s) were also evaluated to determine the potential applicability of *Lpb. plantarum* R23 in the bio-preservation of foods. Despite the long history of several *Lpb. plantarum* strains as probiotics and starter cultures in the food industry, the continuous isolation and study of new potential strains by the scientific community are significant for the improvement of food safety and, at the same time, to continuously track the interests of the consumers.

## 2. Materials and Methods

### 2.1. Origin, Growth, and Storage Conditions of Lactiplantibacillus plantarum R23

*Lactiplantibacillus plantarum* R23 was isolated from pre-washed bagged arugula, screened for antimicrobial activity and bacteriocin production, and identified by whole-genome sequencing in our previous publication [15].

This strain was grown aerobically on de Man, Rogosa and Sharpe (MRS) agar and/or broth (Lab M, Bury, UK) at 30 °C for 24 h and stored at −80 °C in MRS broth (Lab M) containing 30% (*v*/*v*) of glycerol (Sigma, Steinheim, Germany) and sub-cultured twice before use in assays.

### 2.2. Whole-Genome Sequencing and Bioinformatic Analyses

The genome sequence of *Lpb. plantarum* R23 was performed by using whole-genome sequencing (2 × 150 bp) in an Illumina NovaSeq platform. The paired-end fastq files were used to assemble the bacterial genome with the SPAdes genome assembler (version SPAdes-3.12.0-Linux) in a Linux environment [27].

#### 2.2.1. Virulence and Resistance Profiles of *Lpb. plantarum* R23

The ResFinder 4.1 server (accessed on 13 November 2020, at https://cge.cbs.dtu.dk/services/ResFinder/) [28] was used for predictions of resistance phenotypes from the assembled genome.

A search for the presence of virulence factor determinants was performed by blastx alignment of the sequence contigs against the protein sequences of the core dataset VFDB_setA_pro.fas of the Virulence Factors of Pathogenic Bacteria Database VFDB (accessed on 13 November 2020, at http://www.mgc.ac.cn/cgi-bin/VFs/v5/main.cgi) [29] in a Linux environment, using a filter for E-Values < 10^−15^.

#### 2.2.2. Identification of *Lpb. plantarum* R23-Produced Bacteriocin

To identify the bacteriocin produced by *Lpb. plantarum* R23, the fasta file with the generated contigs was used to search for bacteriocins in the core peptide database of bacteriocin classes I, II, and III, on the webserver BAGEL4 (accessed on 13 November 2020, at http://bagel4.molgenrug.nl) [30].

They were also aligned with the BACTIBASE protein fasta file (downloaded on 13 November 2020, at http://bactibase.hammamilab.org/downloads.php), a data repository of natural antimicrobial bacteriocin peptides [31]. The alignment was performed using the blastx tool from the blast (basic local alignment search tool) ncbi-blast-2.10.1 + -1.x86_64.rpm version [32] in a Linux environment, using a filter for E-Values < 10^−15^.

### 2.3. Characterization of Lpb. plantarum R23-Produced Bacteriocin

#### 2.3.1. Kinetics of Growth and Bacteriocin Production

An overnight culture was inoculated (1% *v*/*v* into 150 mL of MRS broth) and incubated at 30 °C, without shaking. Samples were taken at regular intervals during 24 h of incubation. Changes in pH and optical density (600 nm) were recorded every hour, and bacteriocin activity (arbitrary unit (AU)/mL) and viable counts were calculated every 3 h, as described by Van Reenen et al. [33]. *Listeria monocytogenes* strains L7946 and L7947 [34] were used as target organisms. Two independent replicates were performed.

#### 2.3.2. Effect of Enzymes, Temperature, pH, and Surfactants on Bacteriocin Activity

*Lactiplantibacillus plantarum* R23 was grown in MRS broth overnight at 30 °C. Cells were harvested (8000× *g*, 10 min, 4 °C), and the cell-free supernatant (CFS) was adjusted to pH 6.5 with 1 M NaOH. One milliliter sterile CFS was incubated for 2 h in the presence of proteinase K, trypsin, papain, pepsin, α-amylase and catalase (all from Boehringer Mannheim GmbH, Mannheim, Germany) at both 1 mg/mL and 0.1 mg/mL (final concentrations). The remaining antimicrobial activity was monitored by the agar spot test method. In a separate experiment, 1% (*w*/*v*) sodium dodecyl sulphate (SDS), ethylenediaminetetraacetic (EDTA), Tween 20, Tween 80, urea, Triton X-100, Triton X-114, ox-bile, urea, and NaCl were added to bacteriocin-containing CFS. EDTA was added to CFS in final concentrations of 1.0, 2.0 and 5.0 mM. Untreated CFS and detergents, at the respective concentrations in water, were used as controls. All samples were incubated at 30 °C for 5 h and then tested for antimicrobial activity. The effect of pH on bacteriocin activity was tested by adjusting the pH of sterile CFS from 2.0 to 12.0 (at increments of two pH units) with sterile 1M NaOH or 1M HCl. After 1 h of incubation at room temperature (25 °C), samples were readjusted to pH 6.5 with sterile 1 M NaOH or 1 M HCl, heated to 80 °C for 10 min, and tested for antimicrobial activity. The effect of temperature on bacteriocin activity was tested by incubating CFS at 4, 25, 30, 37, 45, 60, 80, and 100 °C for 120 min. Bacteriocin activity was also tested after 15 min at 121 °C. The agar spot test method for the antimicrobial activity [33] was used in all experiments. *Listeria monocytogenes* strains L7946 and L7947 served as target strains.

#### 2.3.3. Cell Lysis

Twenty milliliters of the bacteriocin-containing cell-free supernatant (12,800 AU/mL, pH 6.0 assayed on *L. monocytogenes* L7946 and *L. monocytogenes* L7947) was filter-sterilized and added to 100 mL early exponential phase (5 h old; OD = 0.6) cultures of *L. monocytogenes* L7946 and L7947. Optical density readings at 600 nm were taken every hour for 13 h. Both *L. monocytogenes* cultures without added bacteriocins were used as controls, and two independent replicates were performed.

#### 2.3.4. Adsorption Studies

Adsorption of bacteriocin to producer cells was studied according to the method described by Yang et al. [35]. *Lactiplantibacillus plantarum* R23 was cultured for 18 h at 30 °C. The pH of the culture was adjusted to 6.0 with 1 M NaOH to allow maximal adsorption of the bacteriocin to the producer cells. The cells were then harvested (12,000× *g*, 15 min, 4 °C) and washed with sterile 0.1 M phosphate buffer (pH 6.5). The pellet was re-suspended in 10 mL 100 mM NaCl (pH 2.0) and agitated for 1 h at 4 °C to allow delaminating bacteriocin from the cells. The cells were then harvested (12,000× *g*, 15 min, 4 °C), the cell-free supernatant neutralized to pH 7.0 with sterile 1 M NaOH, and was then tested for bacteriocin activity, as described by Van Reenen et al. [33].

#### 2.3.5. Partial Purification and Molecular Size

After 18 h growth of the *Lpb. plantarum* R23 culture, the supernatant resulting from the first centrifugation (12,000× *g*, 20 min, 4 °C) was kept at 4 °C for partial purification. Ammonium sulphate was added gradually to the stored supernatant to reach 40%, 60%, and 80% of saturation, in independent experiments, and each solution was kept at slow stirring for 4 h at 4 °C. After centrifugation (12,000 rpm, 20 min, 4 °C), precipitated proteins in the pellet and floating on the surface were collected and dissolved in 25 mM ammonium acetate buffer (pH 6.5), following the method described by Sambrook et al. [36]. All samples were stored at −20 °C.

To determine the bacteriocin molecular size, samples were separated by tricine-SDS-PAGE according to Schägger and Von Jagow [37]. A low molecular weight marker with sizes ranging from 6.5 kDa to 270 kDa (GRS Protein Marker MultiColour PLUS; Grisp, Porto, Portugal) was used. After running, the gel was split in two. One half was fixed with 20% isopropanol and 10% acetic acid, and the other half was stained with Coomassie Brilliant Blue R250 (Bio-Rad, Hercules, CA, USA) to visualize the position of the peptide band. The position of the active bacteriocin was determined by overlaying the half of the unstained-gel, and extensively pre-washed with the sterile distilled water, with cells of *L. monocytogenes* L7946 (106 colony-forming units (CFU)/mL), firstly embedded in brain heart infusion (BHI; Biokar, Beauvais, France) soft agar (0.7% agar *w*/*v*).

## 3. Results

### 3.1. Characterization of Lpb. plantarum R23 by Whole-Genome Sequencing

#### 3.1.1. Presence of Antibiotic Resistances

No antibiotic resistance phenotype could be predicted from the analysis of the contigs of *Lpb. plantarum* R23, because the ResFinder 4.1 software could not detect any homologue of genes encoding antibiotic resistance.

#### 3.1.2. Presence of Virulence Factors

No significant blastx alignments (E value < 10^−15^ and 60% of alignment coverage) were detected between the translated DNA sequences of the assembled contigs and the database of pathogenic bacteria virulence factors, such as proteins engaged in adhesion and invasion, secretion systems and its effectors, toxins, and iron acquisition by the bacterial cell.

### 3.2. Identification of Lpb. plantarum R23 Bacteriocin by Whole-Genome Sequencing

**Figure 1:**
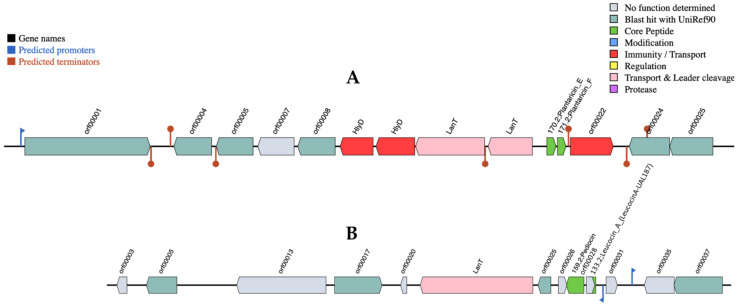
Genetic organization map of the plantaricins (**A**) and pediocin (**B**) gene clusters of the *Lpb. plantarum* R23 strain.

The BAGEL4 software showed that a contig of the assembled bacterial genome encodes two proteins that are 100% identical to the class II bacteriocins, Plantaricin E and Plantaricin F, both from the bacterium *Lpb. plantarum* (Figure 1A). Upstream of the genes encoding plantaricins, and in the same operon, two *lanT* gene homologues encoded the bacteriocin ABC transporter, the ATP binding protein, and the permease protein PlnG.

The sequence of another contig showed 100% identity with the pediocin proteins (Figure 1B) of the *Pediococcus acidilactici* organism. In the same operon, located downstream was the *LanT* gene that encoded the transport-processing Pediocin PA-1 of the ATP-binding protein PedD and an open reading frame (ORF) that encoded the pediocin biosynthesis protein PA-1 P. This operon also included a gene encoding a Leucocin A homologue.

These results were confirmed by the alignment of blastx against BACTIBASE, a database of bacterial antimicrobial peptides. Significant alignments confirmed the presence of the following protein homologues: Plantaricin F in a contig; Pediocin PA-1 (Pediocin ACH), and Coagulin A in the other.

### 3.3. Characterization of Lpb. plantarum R23-Produced Bacteriocin

Based on WGS, *Lpb. plantarum* R23 may produce more than one bacteriocin. All the experiments characterizing R23 bacteriocin were performed using the crude supernatant; therefore, the results presented in the following sections may refer to one or more bacteriocins.

#### 3.3.1. Kinetics of Growth and Bacteriocin Production

**Figure 2:**
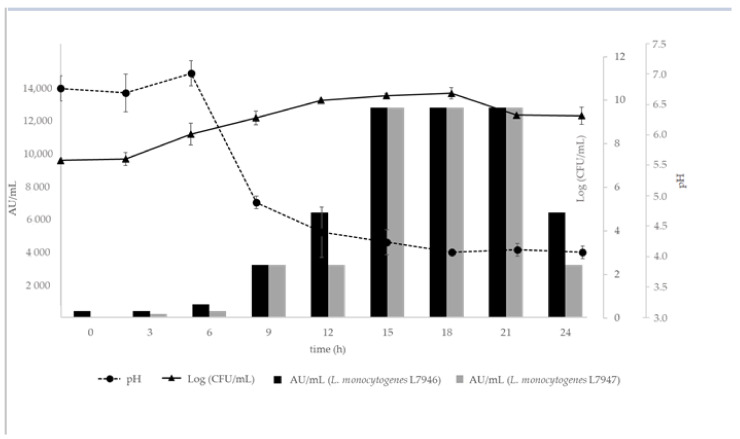
Production of bacteriocin(s) by *Lpb. plantarum* R23 in MRS broth (pH 6.4) at 30 °C. The antimicrobial activity of cell-free supernatants is presented as AU/mL (bars) for *L. monocytogenes* L7946 and L7947 strains.

The bacteriocin(s) activity in AU/mL produced by *Lpb. plantarum* R23 against the two *Listeria* strains over time is shown in Figure 2. It is possible to conclude that the bacteriocin(s) showed maximum activity between 15 and 21 h (AU/mL = 12,800). The pH value started to decrease from 6 h and stabilized after 15 h.

#### 3.3.2. Effect of Enzymes, Temperature, pH, and Surfactants on Bacteriocin(s) Activity

The impact of certain detergents, enzymes, pH, and temperature values on bacteriocin(s) activity against *L. monocytogenes* L7946 and L7947 are shown in Table 1.

The bacteriocin(s) was resistant to most detergents because the reduction in its activity was negligible. For the enzymes tested, only papain and catalase did not decrease bacteriocin(s) activity, while trypsin and proteinase K reduced bacteriocinogenic activity by 100%. As for the pH values, it was possible to verify that the bacteriocin(s) was quite resistant to a wide range of values (between 2 and 8). The same was verified regarding the tested temperatures, with a marked decrease in bacteriocin(s) activity only at 121 °C.

#### 3.3.3. Cell Lysis

**Figure 3:**
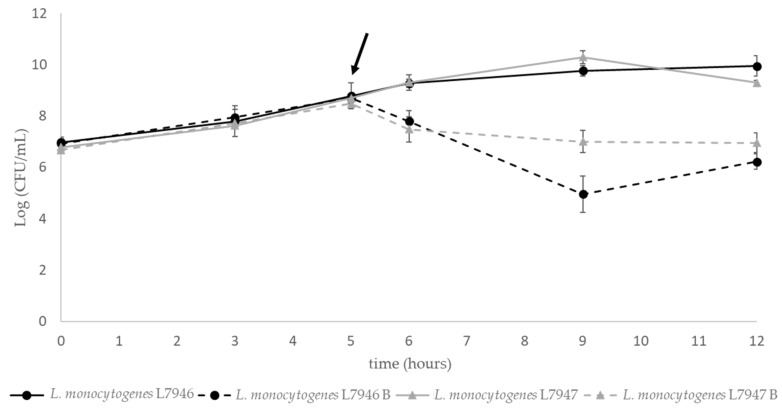
Effect of R23 bacteriocin(s) on the growth of *L. monocytogenes* L9746 (dotted black lines) and *L. monocytogenes* L7947 (dotted grey lines) presented as log (CFU/mL). The continuous line represents target cultures without added bacteriocins. The arrow indicates the point at which the bacteriocin-containing CFS was added.

The inhibitory effect of *Lpb. plantarum* R23 bacteriocin(s) produced over time and the type of pathogen used is shown in Figure 3. It was found that after the addition of the bacteriocin-containing cell-free supernatant to the pathogen at the beginning of the exponential phase (after 5 h of growth), its growth was inhibited.

#### 3.3.4. Adsorption Studies and Molecular Size

The bacteriocin(s) did not adhere to the surface of the producer cells, because its activity was not detected (0 AU/mL) after the treatment of *Lpb. plantarum* R23 with 100 mM NaCl at pH 2.0.

The approximate molecular size of this bacteriocin was below 6.5 kDa (data not shown). When the gel with the peptide band was overlaid with cells of *L. monocytogenes* L7946 already embedded in BHI soft agar, the position of the active bacteriocin was observed by a zone of growth inhibition, which was coincident with the position of the active bacteriocin in the molecular mass marker.

## 4. Discussion

*Lactiplantibacillus plantarum* R23 was isolated from arugula and was first screened for bacteriocin production and probiotic potential in one of our previous studies [15]. In the absence of a protector food matrix, this strain could not survive through the simulated digestion, making its probiotic potential unfeasible. However, this strain’s ability to produce a bacteriocin with activity against deteriorating and pathogenic microorganisms (*Bacillus subtilis*, *Enterococcus faecalis*, *Listeria innocua*, and *L. monocytogenes*) is a great advantage that should not be ignored. Thus, this study arose intending to study the characteristics of the *Lpb. plantarum* R23 strain and its produced bacteriocin(s) to ascertain its potential to be used in the food industry as a bio-preservative or starter culture.

The results from the analysis of *Lpb. plantarum* R23 contigs may suggest that this genome does not contain any antibiotic resistance genes. However, because the assembled genome is not closed, the presence of any antibiotic-resistant gene encoded in any uncovered region of the bacterial genome cannot be excluded. On the other hand, resistance may also result from changes/mutations in some genes codifying proteins involved in resistance, an aspect that was not addressed during this study. Nevertheless, in our previous study [15], it had already been proven that the *Lpb. plantarum* R23 was inhibited by the established antibiotics at concentrations equal to or lower than the cut-off values defined by the Panel on Additives and Products or Substances in Animal Feed (FEEDAP) of the EFSA [38]. Both phenotypic susceptibility and the absence of transferable antimicrobial resistance genes suggest that *Lpb. plantarum* R23 is considered acceptable to be used as a feed additive [38].

Despite some genes encoding virulence factors possibly being located in an un-sequenced region of the genome, the absence of detectable virulence factors from the assembled contigs suggests that *Lpb. plantarum* R23 does not have classical virulence factors. This is also corroborated by its phenotypic and genotypic characterization in our previous study; biogenic amines, hemolysis, gelatinase, and DNase were not produced by this strain, and virulence determinants screened by PCR assays were not detected [15].

Several authors have reported the isolation of other *Lpb. plantarum* strains from various foods and their safety due to the absence of acquired antibiotic resistance genes [39,40] and virulence factors [39,41]. The identification and characterization of *Lpb. plantarum* R23 by whole-genome sequencing confirms the results previously obtained during the characterization of this bacterium [15]. These characteristics indicate that *Lpb. plantarum* R23 may be considered a safe culture to be used in the food industry. According to a report published by the EFSA [42], the whole-genome sequencing analysis should be performed for all the strains intentionally used in the food chain, because it can provide unequivocal information regarding their taxonomic identification and their potential virulence factors and resistance to antibiotics of clinical relevance.

As previously mentioned, *Lpb. plantarum* R23 demonstrated antimicrobial activity due to its bacteriocinogenic activity against Gram-positive pathogens and spoilage bacteria [15]. From the whole-genome sequencing analysis, two 100% identical proteins to the class II bacteriocins—Plantaricin E and Plantaricin F—were found (Figure 1A). Plantaricin E is a bacteriocin precursor of the PlnE peptide [43], and the other ORFs of this operon share homology with *plnD*, *plnG*, *plnH* and *plnS*, suggesting that they belong to a *pln* operon, such as the one involved in the production of bacteriocin from *Lpb. plantarum* [44]. Besides plantaricin, the other contigs Pediocin PA-1 (Pediocin AcH) and Coagulin A (Figure 1B) were also found to share 100% identity with the Pediocin proteins. Coagulins are anti-*listeria* bacteriocins belonging to the Pediocin family [45].

Plantaricins and Pediocins belong to a class II bacteriocins, a large group of small (<10 kDa), heat-stable, and non-lantibiotic peptides [46]. Pediocin PA-/AcH is one of the most studied class II bacteriocin and its production by different *Lpb. plantarum* strains has already been reported [47,48]. Additionally, several plantaricins produced by several *Lpb. plantarum* strains have been found and characterized [49,50,51,52].

The bacteriocin(s) produced by *Lpb. plantarum* R23 was semi-purified and characterized in this study. It is important to highlight that the R23 bacteriocin(s) characterization was performed using the crude supernatant, thus it will be necessary to further purify and identify the peptide(s).

To elucidate the time of incubation at which *Lpb. plantarum* R23 exhibited the maximum bacteriocin(s) production, the profiles of the growth kinetics and bacteriocin(s) activity in AU/mL against two *Listeria* strains over time were determined (Figure 2). The decrease in pH values over time (after 6 h and stabilization after 15 h) is due to the lactic acid produced by *Lpb. plantarum* R23. The maximum bacteriocin(s) activity (AU/mL = 12,800) occurred between 15 and 21 h of *Lpb. plantarum* R23 growth, which is in accordance with what has been reported by other authors [53,54]. The supernatant of the *Lpb. plantarum* R23 cultured for 18 h was therefore used for subsequent experiments. The bacteriocin(s) production in the early stages of *Lpb. plantarum* R23 growth cycle is a considerable advantage when competitive growth is desired or for the inhibition of pathogens before reaching their exponential growth phase.

The stability of the bacteriocin(s) against two *L. monocytogenes* strains when exposed to different treatments with enzymes, temperatures, pH values, and surfactants was evaluated (Table 1).

The antimicrobial activity of the bacteriocin(s) produced by *Lpb. plantarum* R23 was affected (12.5–100% reduction) at pH values above 8. At pH values of 2 and 8, the reductions were very low (1.56%). This suggests that this bacteriocin(s) is sensitive to alkaline environments, as already verified for other *Lpb. plantarum*-produced bacteriocins [55,56].

The bacteriocin(s) activity was maintained at temperatures between 4 and 100 °C, which corroborates its thermo-stability, one of the characteristics of bacteriocins belonging to class II bacteriocins. Negligible reductions were observed below 30 °C and above 37 °C, and no activity (100% of reduction) was only observed at 121 °C. Similar results were also obtained for other bacteriocins produced by different *Lpb. plantarum* strains [56,57].

A slight reduction in bacteriocin activity(s) was observed for most detergents tested, except for Triton X-100 and Triton X-114 at 0.01 g/mL (50% reduction). Only for catalase and α-amylase enzymes was the bacteriocin activity not fully reduced, confirming its proteinaceous nature. Other authors have already reported similar bacteriocin stabilities after the treatment of the same detergents and/or enzymes [53,56,58].

Another important feature is the effect of the bacteriocin on the target microorganism’s growth, evaluating their potential cell lysis. When the supernatant of *Lpb. plantarum* R23 was added to a 5 h old culture of *L. monocytogenes* (early exponential phase), decreases of about two log cycles were observed for both target strains (Figure 3). However, after 9 h and until 12 h of incubation, a slow growth of both *L. monocytogenes* strains was observed. Apparently, the bacteriocin(s) had an effect on the pathogen viability at the moment of addition, but were reduced over time. This means that bacteriocin(s) produced by *Lpb. plantarum* R23 had a bacteriostatic mode of action. Although a vast number of *Lpb. plantarum* produced bacteriocins with bactericidal effect [56,59,60], a similar bacteriostatic effect has been reported for other bacteriocins [61,62,63].

Bacteriocin(s) produced by *Lpb. plantarum* R23 did not adsorb to the producer cells, because its activity was not detected after treatment of *Lpb. plantarum* R23 with 100 mM NaCl at pH 2.0. Similar observations were also recorded for Plantaricin ST31 [64] and Pediocin ST194BZ [65].

Finally, as expected, the molecular size of the *Lpb. plantarum* R23 bacteriocin(s) was below 6.5 kDa, as determined by tricine-SDS-PAGE (Figure S1). This is in agreement with the identification obtained by whole-genome sequencing because plantaricins and pediocins are small peptides (<10 kDa) belonging to class II bacteriocins. This molecular size is also within the range of most bacteriocins reported for other *Lpb. plantarum* strains [53,66,67].

## 5. Conclusions

From both phenotypic characterization and whole-genome sequencing analysis, *Lpb. plantarum* R23 may be considered a safe culture to be used in the food industry. Additionally, this bacterium produces a stable bacteriocin which, besides being safe, makes it a potential bio-preservative culture inhibiting pathogens such as *L. monocytogenes* and spoilage bacteria to guarantee the safety and the extension of food shelf life. Once *Lpb. plantarum* R23 harbors genes encoding for the production of Plantaricin E, Plantaricin F, Pediocin PA-1 (Pediocin AcH), and Coagulin A, recognition of the conditions in which more than one bacteriocin, or even all, are produced is mandatory to increase knowledge about *Lpb. plantarum* R23 applicability.

After the preliminary characterization of *Lpb. plantarum* R23, it is possible to conclude that it is a relevant strain with promising characteristics, which should be studied more deeply to evaluate its potential future use in the food industry as a bio-protective or starter culture.

## Figures and Tables

**Table 1:** Effect of different treatments on bacteriocin(s) activity against the target organisms *L. monocytogenes* L7946 and *L. monocytogenes* L7947, expressed in corresponding percentage values (%) of reduction.

		*L. monocytogenes* L7946	*L. monocytogenes* L7947
pH	2	1.6%	1.6%
	4	0.0%	0.0%
	6	0.0%	0.0%
	8	1.6%	1.6%
	10	12.5%	12.5%
	12	100.0%	100.0%
Temperature (°C)	4	3.1%	3.1%
	25	1.6%	1.6%
	30 and 37	0.0%	0.0%
	45	3.1%	6.3%
	60	3.1%	6.3%
	80	6.3%	6.3%
	100	12.5%	12.5%
	121	100.0%	100.0%
Enzymes (mg/mL)	Proteinase K_1.0and0.1_	100.0%	100.0%
	Papain_1.0_	50.0%	50.0%
	Papain_0.1_	50.0%	50.0%
	Pepsin_1.0_	100.0%	100.0%
	Pepsin_0.1_	50.0%	25.0%
	Trypsin_1.0and0.1_	100.0%	100.0%
	α-amylase_1.0_	6.3%	25.0%
	α-amylase_0.1_	0.0%	0.0%
	Catalase_1.0and0.1_	0.0%	0.0%
Detergents	Tween 20 and Tween 80_0.01g/mL_	3.1%	3.1%
	Triton X-114 and Triton X-100_0.01g/mL_	50.0%	50.0%
	SDS_0.01g/mL_	0.0%	1.6%
	EDTA_0.1mM_	3.1%	3.1%
	EDTA_2.0mM_	3.1%	3.1%
	EDTA_5.0mM_	6.3%	6.3%
	Ox-bile_0.01g/mL_	1.6%	1.6%
	Urea and NaCl_0.01g/mL_	0.0%	0.0%

Note: Without treatment, the maximum activity of R23 bacteriocin(s) for each *L. monocytogenes* strain was 12,800 AU/mL. The percentage values of inhibition are represented according to this maximum activity.

## Data Availability

Not applicable.

## Supplementary Materials

The following are available online at https://www.mdpi.com/article/10.3390/ijerph18115515/s1. Figure S1: Tricine-SDS Page of Bacteriocin(s) produced by Lpb. plantarum R23. Legend: Lanes 1 to 3: peptide bands stained with Coomassie Blue R250 with 40% saturated ammonium sulfate (lane 1); 60% saturated ammonium sulfate (lane 2); 80% saturated ammonium sulfate (lane 3); lane 4: molecular mass marker; lane 5: zone of growth inhibition, corresponding to the position of the peptide bands in lane 1; lane 6: zone of growth inhibition, corresponding to the position of the peptide bands in lane 2 and lane 7: zone of growth inhibition, corresponding to the position of the peptide bands in lane 3.
