# Supplementary figures and images for: Characterization of a Lactiplantibacillus plantarum R23 Isolated from Arugula by Whole-Genome Sequencing and Its Bacteriocin Production Ability
Source: Int J Environ Res Public Health. 2021 May 21;18(11):5515. doi: 10.3390/ijerph18115515 (PMC8196627; doi:10.3390/ijerph18115515)

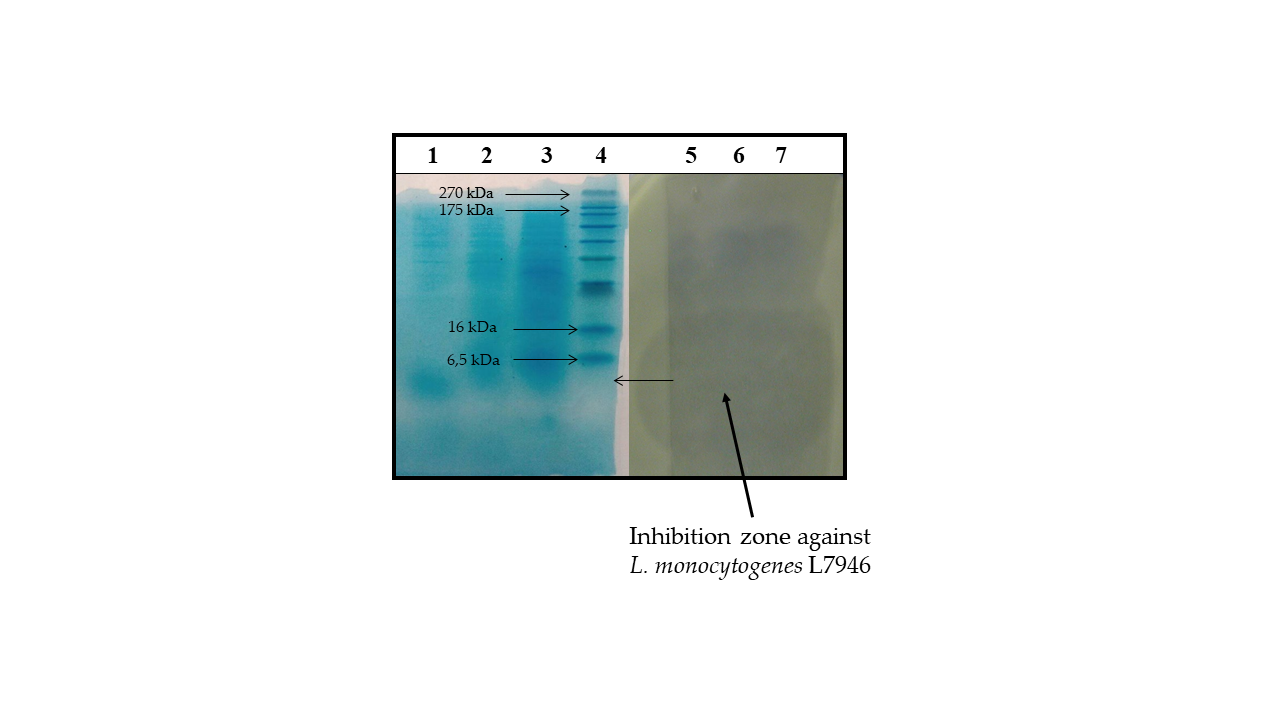

Supplement: Supplementary file 1 [file ijerph-18-05515-s001.zip › ijerph-1205176-supplementary.tif]
